# Inhibiting Epileptiform Activity in Cognitive Disorders: Possibilities for a Novel Therapeutic Approach

**DOI:** 10.3389/fnins.2020.557416

**Published:** 2020-10-15

**Authors:** Andras Attila Horvath, Emoke Anna Csernus, Sara Lality, Rafal M. Kaminski, Anita Kamondi

**Affiliations:** ^1^Department of Anatomy, Histology and Embryology, Semmelweis University, Budapest, Hungary; ^2^Department of Neurology, National Institute of Clinical Neurosciences, Budapest, Hungary; ^3^Department of Radiology, Semmelweis University, Budapest, Hungary; ^4^Faculty of Medicine, Semmelweis University, Budapest, Hungary; ^5^Department of Medicinal Chemistry, Faculty of Pharmacy, Jagiellonian University Medical College, Krakow, Poland; ^6^Department of Neurology, Semmelweis University, Budapest, Hungary

**Keywords:** neurocognitive disorder, epileptiform activity, electroencephalography, cognitive decline, memory consolidation, antiepileptic drugs

## Abstract

Cognitive impairment is a common and seriously debilitating symptom of various mental and neurological disorders including autism, attention deficit hyperactivity disorder, multiple sclerosis, epilepsy, and neurodegenerative diseases, like Alzheimer’s disease. In these conditions, high prevalence of epileptiform activity emerges as a common pathophysiological hallmark. Growing body of evidence suggests that this discrete but abnormal activity might have a long-term negative impact on cognitive performance due to neuronal circuitries’ remodeling, altered sleep structure, pathological hippocampo–cortical coupling, and even progressive neuronal loss. In animal models, epileptiform activity was shown to enhance the formation of pathological amyloid and tau proteins that in turn trigger network hyperexcitability. Abolishing epileptiform discharges might slow down the cognitive deterioration. These findings might provide basis for therapeutic use of antiepileptic drugs in neurodegenerative cognitive disorders. The aim of our review is to describe the data on the prevalence of epileptiform activity in various cognitive disorders, to summarize the current knowledge of the mechanisms of epileptic activity in relation to cognitive impairment, and to explore the utility of antiepileptic drugs in the therapy of cognitive disorders. We also propose future directions for drug development and novel therapeutic interventions targeting epileptiform discharges in these disorders.

## Introduction

Cognitive impairment is a common symptom of various neurological and psychiatric disorders including autism spectrum disorder (ASD), schizophrenia, attention deficit hyperactivity disorder (ADHD), multiple sclerosis (MS), and major neurocognitive disorders (NCDs). The cumulative prevalence of these conditions is ∼50% in developed societies creating prominent medical and social burden. While the mentioned diseases differ significantly in their symptoms and pathological background, diminished memory function is a common characteristic.

The association between epilepsy and the above-mentioned diseases evoked a remarkable interest in the medical literature highlighting various hypotheses and explanations of bidirectional connections. Seemingly, all these syndromes increase the risk for epileptic seizures.

In ASD, reports agree that patients have increased incidence for epileptic seizures ranging from 5 to 38% (Hara, 2007). Symptoms of ADHD are highly common in children affected by epilepsy, and epilepsy is predominantly associated with inattentive type of ADHD (Plioplys et al., 2007). Other studies proposed that children with attention problems have a two–threefold increase for unprovoked seizure occurrence (Austin and Caplan, 2007).

Recently, it has been established that epilepsy is a frequent comorbidity in various forms of NCD (Horváth et al., 2016). Studies on familial AD steadily demonstrate that seizures affect approximately half of patients (Zarea et al., 2016). A study of Beagle et al. demonstrated ∼15% cumulative probability of developing seizures by patients with diffuse Lewy-body dementia (DLB) and 3% by patients with frontotemporal degeneration (FTD) (Beagle et al., 2017). Furthermore, epileptic patients also have a higher chance for late life neurocognitive disorders (Subota et al., 2017).

While numerous studies investigated the link between epileptic seizures and cognitive disorders, reports on epileptiform activity between seizures [interictal epileptiform activity (IEA)] or without seizure activity [subclinical epileptiform activity (SEA)] are underrepresented. While classic epileptology focused on the accurate control of seizures, in recent years, growing body of evidence suggests that IEA might have harmful effect on cognitive functions (Glennon et al., 2016; Hu et al., 2016; Meekes and Jennekens-Schinkel, 2018). It is intriguing to analyze the potential role of SEA as well, since SEA shows similar electrographic features as IEA and the above-mentioned cognitive disorders share another hallmark: the prevalence of SEA is elevated in all conditions.

The aim of our opinion review is to describe the results of studies on the prevalence of IEA and SEA in the various forms of cognitive disorders, to summarize the current knowledge on the effect of epileptiform discharges on cognitive functions, and to propose new directions for therapeutic interventions targeting cognitive decline. To increase the accuracy and novelty of our research, we analyzed studies published later than 2000, and in the prevalence and therapy sections, we included reports on humans only.

## Prevalence of SEA and IEA in Cognitive Disorders

### Major Neurocognitive Disorders

NCDs represent 80–100 various conditions with progressive neurodegenerative process. AD is the leading cause of cognitive decline by the elderly affecting 37.5 million people worldwide, and this number is expected to triple by 2050 (Abbott, 2011). The first symptoms of AD—as the impairment of episodic memory and difficulty in spatial orientation—occur usually at age of 60–70. During the 6–8 years of disease course, patients lose other cognitive skills including orientation, communication, and language skills and finally the ability of self-care (Cummings and Cole, 2002). The pathological hallmark of AD is the accumulation and progressive spread of misfolded amyloid and tau proteins (Ittner and Götz, 2011). Since we are not able to significantly slow down the progression of cognitive deterioration (Cummings and Cole, 2002), there is a clear need to find possibly modifiable factors of AD, especially in the early phases of the disease. A recently recognized contributor to AD progression is epileptic activity. Numerous human studies highlighted that AD patients have a higher chance to develop epileptic seizures (Horváth et al., 2016). IEA was analyzed in three studies with routine electroencephalogram (EEG) identifying interictal epileptiform activity in third of AD patients who presented with epileptic seizure (Rao et al., 2009; Cretin et al., 2016; Sarkis et al., 2016). In two sleep EEG studies, IEA rate was 62% (Vossel et al., 2013) and 80% (Horváth et al., 2018b) in patients with clinical history of seizures. In these studies, IEA appeared mainly over the frontotemporal areas with a left-side dominance (Rao et al., 2009; Vossel et al., 2013; Cretin et al., 2016; Sarkis et al., 2016; Horváth et al., 2018b). Temporal occurrence of IEA was analyzed only in two studies: in the study of Vossel et al. (2013), 10% of IEA was detected during wakefulness and 64% appeared exclusively in stage2 or deeper sleep, while in our previous report, 82% of IEA was associated with sleep and 55% was detected in deep sleep (Horváth et al., 2017b).

There are only a few studies analyzing the occurrence of SEA in AD. Liedorp at al. found epileptiform discharges in only 3% of 1,674 AD patients (Liedorp et al., 2010) using 30 min long daytime EEGs. Vossel et al. revealed SEA in 6% of 113 AD and MCI patients evaluating daytime routine EEGs in 91% and serial or long-term EEGs in 7% of the patients (Vossel et al., 2013). In another study of Vossel et al. using magnetoencephalography and sleep EEG, SEA was found in 42% of AD patients who have never experienced epileptic seizure before (Vossel et al., 2016). They analyzed the temporal distribution of SEA as well showing that epileptic activity occurs almost completely (90%) during sleep and mainly over the temporal regions. This is in line with our previous reports showing the important role of sleep EEG in the detection of SEA in AD (Horváth et al., 2017b, 2018a). It should be noted that in Vossel’s study from 2016, SEA was associated with faster deterioration of cognition determined by Mini-Mental Score Examination (Vossel et al., 2016). Moreover, studies also suggest that AD and mild cognitive impairment (MCI) patients with SEA have an earlier onset of cognitive decline being usually associated with more aggressive forms of AD that show faster progression (Vossel et al., 2016; Horváth et al., 2018b). Table 1 summarizes the AD studies on the prevalence of IEA and SEA.

**TABLE 1:** Prevalence of epileptiform discharges in Alzheimer’s disease.

References	N	Study design	EEG-type	ED (%)
Rao et al., 2009	39	Retrospective, epileptic AD patients	Routine (74%) or no EEG (26%)	38% (IEA)
Cretin et al., 2016	13	Retrospective, epileptic MCI patients	Routine	100% (IEA)
Sarkis et al., 2016	77	Retrospective, epileptic AD patients	Routine	22% (IEA)
Vossel et al., 2013	54	Retrospective, MCI + AD patients	Routine and serial	62% (IEA), 6% (SEA)
Horváth et al., 2018b	42	Prospective	24 h	20% (IEA), 28% (SEA)
Liedorp et al., 2010	1,674	Retrospective	–	3% (SEA)
Vossel et al., 2016	33	Prospective, non-epileptic AD patients	24 h + magnetoencephalography	42% (SEA)

*N, number of patients; MCI, mild cognitive impairment; ED, epileptiform discharge; IEA, interictal epileptiform activity; SEA, subclinical epileptiform activity*

DLB is the second most common type of dementia accompanied by changes in behavior, cognition, movement, sleep, and the autonomic functions (Savica et al., 2013). The major symptoms are the rapid eye movement sleep (REM) sleep behavior disorder, memory loss, and visual hallucinations (McKeith, 2002). Furthermore, marked fluctuations in attention or alertness, parkinsonism (slowness of movement, troubled walking, or rigidity), and dysfunction of autonomic nervous system (orthostatic hypotonia, constipation) are also present (McKeith, 2002). An important diagnostic hallmark is the hypersensitivity for antipsychotic drugs (McKeith, 2002). The major pathological finding is the widespread accumulation of alpha-synuclein protein (Hishikawa et al., 2003). Reports on DLB-related epilepsy are less frequent compared to AD; however, a recent paper depicts that DLB patients are susceptible for seizures similarly to AD patients (Beagle et al., 2017). Another study using postmortem approach identified myoclonus with the retrospective analysis of clinical records in 21.7% of DLB patients, and it was associated with earlier onset of cognitive decline (Morris et al., 2015). While reports on IED or SEA in DLB are absent, considering that DLB patients might have a similar prevalence of seizures than AD patients, analyzing IED/SEA in DLB is an important future direction.

FTD is a heterogeneous condition encompassing five types of dementia including behavior and language-dominant lobar degenerations (behavioral variant, semantic variant primary progressive aphasia, and non-fluent variant primary progressive aphasia) and motor dominant disorders (corticobasal syndrome, progressive supranuclear palsy) (Bang et al., 2015). Initial symptoms usually appear by adults in their fifth or sixth decade of life (Bang et al., 2015). The histological finding is the progressive accumulation of tau, tdp-43, and fus proteins (Bang et al., 2015). FTD patients tend to have also higher risk for epileptic seizure (Beagle et al., 2017; Arnaldi et al., 2020); however, there is only one case in the literature focusing on the importance of epileptic activity in FTD. With the help of foramen ovale electrodes, SEA was detected in a seizure-free patient with FTD that could have caused the daily variability in her cognitive behavior (Horváth et al., 2017a). Since the number of reported cases on IEA/SEA on FTD is still small, further investigation is necessary (Chan et al., 2004).

Huntington disease is an autosomal dominant neurodegenerative disorder that is characterized by involuntary movements, cognitive decline, and personality changes (Bates et al., 2015). Reports on patients with adult onset showed that prevalence of seizures is similar to the general population (Sipilä et al., 2016). However, epileptic seizures and epileptiform activity occur in 30–40% of patients in the rarer juvenile type (J-HD), which appears in young persons under 21 years of age (Cloud et al., 2012). Currently, there are only a few studies in the literature solely investigating SEA or IEA in Huntington disease. A review of Landau and Cannard (2003) analyzed 23 previously published cases of J-HD patients. Epileptiform abnormalities were noted in 17 (74%). In 10 cases, they were associated with overt epileptic seizures, so the prevalence of IEA was 44%. In seven cases (30%), SEA was detected. Nine patients showed generalized discharges having polyspike and wave activity, while eight others had focal or multifocal epileptiform discharges with posterior predominance. The limitation of this study is that the diagnosis of J-HD was not genetically confirmed. Another study analyzed the pattern of IEA of a J-HD patient with epileptic seizures and described the occipital intermittent rhythmic delta activity as the major hallmark of epileptic activity (Ullrich et al., 2004).

To conclude, patients with various forms of NCD tend to be more vulnerable for epileptic seizures, however, prevalence data show high variability. While SEA is detectable in approximately 17% of AD patients, studies on other NCD forms are scarce. The role of SEA in the accelerated progression of AD draws attention to the need for further investigations.

### Multiple Sclerosis

MS is a heterogeneous demyelinating disease of the central nervous system involving inflammatory processes not only of the white matter but also the juxtacortical and cortical areas. Recent studies also highlight that MS should be also considered as a neurodegenerative disorder (Ziemann et al., 2011). Attention has been mostly focused on clinical seizures, as seizures might occur at any stage of MS. Sponsler and Kendrick-Adey conducted the most extensive review on assessing prevalence of seizures among MS patients by compiling results of 25 scientific papers (Sponsler and Kendrick-Adey, 2011). They found that about 2% of MS patients experienced seizures. A study of 36 patients found that early-onset MS frequency was significantly higher in patients with epileptic seizures as compared to those without epilepsy (Durmus et al., 2013). Epileptic events might be a consequence of edema surrounding the lesions, disease-modifying drugs lowering the epileptic threshold, or the reduced cortical thickness as a result of disease course (Geurts et al., 2005; Cheng et al., 2012; Calabrese et al., 2017). A study by Calabrese et al. (2008) reported intracortical lesions in 90% of epileptic patients with relapsing–remitting MS (RRMS), whereas only in 48% with RRMS without epilepsy. In another study by his group, the most affected gray matter lesions in RRMS epileptic patients were the hippocampus (14.2%), the lateral temporal lobe (13.5%), the cingulate (10.0%), and the insula (8.4%) (Calabrese et al., 2017). Lund et al. suggested that epilepsy in MS should be classified as symptomatic focal epilepsy due to the nature of cortical lesions (Koch et al., 2008; Lund et al., 2014).

Available data on prevalence and background of IEA and SEA in multiple sclerosis are limited. SEA could potentially be a major reference point in guiding a clinician, however, no studies exist that focus solely on SEA in MS patients. EEG abnormalities reported in MS can be diffuse asynchronous theta activity, synchronous rhythmic slow waves, focalized flattened EEG patterns (Striano et al., 2003), or less frequently periodic lateralized epileptiform discharges, which are mostly seen in acute exacerbations of the disease (Lawn et al., 2001; Nyquist et al., 2001; Gandelman-Marton et al., 2003). Table 2 lists some of the studies that looked at EEG abnormalities distinguishing based on epileptiform and non-epileptiform pathological EEG events. However, most of these studies had varying methodology and looked at alterations in MS patients who already were known to have at least one seizure when they all analyzed IEA. IEA was found in 3.9–86.9% of the patients representing the great variability of the study methods (e.g., EEG technique and length of recording, retrospective vs. case–control studies, sample sizes of 23 patients vs. 29,165 patients). Only three studies analyzed SEA independently, suggesting ∼7–8% prevalence. Bustuchina postulated a bidirectional relation between MS and epileptic activity and suggested that MS might be a network disease, and so emphasis should be put on both entities for best therapeutic outcome (Bustuchina Vlaicu, 2019).

**TABLE 2:** Prevalence of epileptiform discharges in multiple sclerosis.

References	N	Study design	EEG type	ED (%)
Dagiasi et al. (2018)	62	Retrospective	Routine	38 (IEA)
Benjaminsen et al. (2017)	431	Retrospective	Routine	3,9 (IEA)
Calabrese et al. (2017)	23	Case–control	Routine	86,9 (IEA)
Kelley and Rodriguez (2009)	168	Review	–	32,7 (IEA)
Nyquist et al. (2001)	43	Retrospective	Sleep–awake	44.2 (IEA)
Sponsler and Kendrick-Adey (2011)	29,164	Review	–	1.95% (SEA)
Cheng et al. (2012)	93	Retrospective	Routine	8.6% (SEA)
Lund et al. (2014)	364	Retrospective	Routine	7.4% (SEA)

*N, number of patients; ED, epileptiform discharge; IEA, interictal epileptiform activity; SEA, subclinical epileptiform activity.*

### Autism Spectrum Disorder

ASD is an umbrella term for several neurodevelopmental conditions defined by the Diagnostic and Statistical Manual of Mental Disorders, fifth edition (DSM-V) classification, which share clinical manifestations in varying degrees. Such manifestations are impairment in sociability, communication deficits, non-verbal interaction issues, restricted range of interest, repetitive behavior, and impairment of intellectual and behavioral flexibility (Tuchman and Rapin, 2002; American Psychiatric Association, 2013). Pathophysiological background of this heterogeneous syndrome originates in the neural circuit disconnection between the association cortex of the frontal lobe and the higher-order multimodal temporal lobe (Assaf et al., 2010; Belger et al., 2011). SEA and IEA might be one of the biomarkers of malfunction of these involved intrinsic connectivity networks. Table 3 summarizes studies that assessed epileptiform discharges in patients diagnosed with ASD. Prevalence of epileptiform activity is reported in 21–75% of patients. Epilepsy has also been associated with ASD, with a rate of 5–39.2% (Hara, 2007; Ghacibeh and Fields, 2015). A study by Clarke et al. found that 32% of their epileptic subjects met the criteria of ASD, however, authors used questionnaires only, and confirming clinical diagnostic tests were not applied (Clarke et al., 2005).

**TABLE 3:** Prevalence of subclinical epileptiform activity (SEA) in autism spectrum disorder (ASD).

References	N	Study design	EEG-type	ED (%)	Localization
Hughes and Melyn (2005)	59	Case–control	Routine + photic stim	75	Generalized, 59% bilateral spikes and 54% slow-wave complexes
Kim et al. (2006)	32	Prospective cohort	Video-EEG	59	Focal/multifocal sharp waves, generalized paroxysmal fast activity
Hrdlicka et al. (2004)	77	Prospective cohort	Polysomnography	38.1	–
Akshoomoff et al. (2007)	60	Prospective cohort	Routine	32	–
Yasuhara (2010)	1014	Prospective cohort	Routine polysomnography	85.8	Frontal spikes 65.6%, multifocal spikes < 10%
Gennaro Nicotera et al. (2019)	69	Routine	Routine	26.08	Focal spikes, 55.55%; multifocal and diffuse spikes, 44.44%
Mulligan and Trauner (2014)	101	Retrospective	Routine	59.4	–
Giannotti et al. (2008)	104	Prospective cohort	Routine polysomnography + photic stim	40.55	–
Hara (2007)	130	Retrospective follow-up	Routine	21	–
Chez et al. (2006)	889	Retrospective	24-h	60.7	Right temporal spikes, 21.5%; bilateral temporal spikes, 20.2%; generalized spike wave, 16.2%
Elsayed and Sayyah (2012)	47	Case–control	Routine	51.1	Focal frontal, occipital, temporal spikes
Hartley-McAndrew and Weinstock (2020)	123	Retrospective	Routine	30	–

*N, number of patients; ED, epileptiform discharge.*

Several studies have suggested that increasing severity of autistic symptoms may be associated with higher likelihood of epileptic abnormalities (Elsayed and Sayyah, 2012; Mulligan and Trauner, 2014). EEG abnormalities have also been associated with autistic regression, lower intellect, delayed motor, and social development in the first year of life (Hrdlicka et al., 2004). This hypothesis is supported by Nicotera et al. as well (Gennaro Nicotera et al., 2019). In their study, epileptiform discharges were also significantly associated with hyperactivity, aggressive behaviors, self-harm behavior, and severe language impairment. Giannotti specifically investigated sleep patterns of ASD children and found that 64.42% of the patients had active sleep problems and also that disrupted sleep was associated with more severe disease course (Giannotti et al., 2008). Regarding the prevention of the syndrome, a 10-year follow-up study conducted by Hara showed that although 18% of the non-epileptic group exhibited SEA on EEG, 68% of epileptic group revealed SEA findings before the onset of epilepsy (Hara, 2007). He suggested that routine EEGs could predict developing epilepsy in the future.

When we consider treating SEA, Chez et al. found that regimental administration of valproic acid normalized the EEG in 46.6% of ASD diagnosed with SEA (Chez et al., 2006). However, we lack studies on EEG changes of ASD patients following ASD therapy, and studies on behavioral aspects could not prove that use of anticonvulsants provided better outcome than placebo (Hirota et al., 2014).

Based on the above, we are still not confident what SEA means on an EEG regarding pathodevelopment of ASD patients, but there are correlations and associations made. Currently EEG screening and prophylactic anticonvulsant treatment is not recommended in ASD (Swatzyna et al., 2019), as we are not certain about the clinical importance of these epileptiform alterations seen on EEG and how clinical outcome would be affected by such medication regime. However, clinicians could consider obtaining a longer EEG examination and overnight EEG video monitoring. Certainly, applying long-term EEG is crucial, as Chez et al. showed that 5% of EEG abnormalities may have been missed in patients who had a negative, routine EEG previously. Chez et al. (2006) and Gennaro Nicotera et al. (2019) found that, when present, EEG abnormalities were detectable predominantly during sleep. For quality assessment prospective, randomized trials are needed, with clear methodology, and with choices of instrumentation that maximize the amount of data gained from the study population.

### ADHD

ADHD is a syndrome defined by the American Psychiatric Association DSM-V as a persistent pattern of inattention and/or hyperactivity–impulsivity that interferes with functioning or development. In its presentation, we distinguish predominantly hyperactive–impulsive, inattentive, or combined subtypes (American Psychiatric Association, 2013). Worldwide the syndrome affects around 5% of children and 2.5% of adults (Polanczyk et al., 2014). EEG and functional imaging research on anatomical aspect of the disease shows involvement of the frontal cortex (Parisi et al., 2010; Schulz et al., 2012; Zaimoglu et al., 2015), particularly the dorsal anterior cingulate cortex manifested by decreased function of this brain area during inhibitory task control (Bush et al., 2005) and EEG paroxysmal abnormalities (Kanemura et al., 2013). Data suggest a pathophysiological and comorbid overlap between ADHD and epilepsy (Dunn and Kronenberger, 2006; Kaufmann et al., 2009; Salpekar and Mishra, 2014), a study of 76 children with epilepsy found that 31% of them had ADHD compared to 6% in the healthy control group (Hermann et al., 2007). Some studies found that in epileptic children, inattentive subtype is dominating while the combined type in those without epilepsy (Dunn and Kronenberger, 2006; Hermann et al., 2007; Socanski et al., 2010; Kanemura et al., 2013); however, others failed to show such relation (Lee et al., 2016). Although there is a challenge of distinguishing EEG abnormalities of ADHD and epilepsy in the same patient, there has been emerging focus on investigating SEA and their relation to transient cognitive impairment in this subgroup of children (Aldenkamp and Arends, 2004; Schubert, 2005).

Table 4 summarizes the prevalence of SEA among studies, which varies from 4.9 to 53.1%. Most studies are retrospective and used routine EEG. Epileptic activity in ADHD is commonly detected as generalized 3-Hz spike-and-wave discharges and paroxysmal abnormalities such as focal spikes (frontal, midtemporal, rolandic or parietal, occipital) (Holtmann et al., 2003; Schubert, 2005; Kanemura et al., 2013). A review by Salpekar et al. pointed out that an increase in theta waves in frontal regions seems to be a consistent EEG abnormality in this subgroup of patients and that alpha wave asymmetry and higher theta-to-beta ratio have also been reported (Salpekar and Mishra, 2014). The effect of antiepileptic drugs (AED) in patients with SEAs seems to show behavioral improvement in those children with frontal spikes but less so in case of the age-dependent Rolandic spike abnormalities (Holtmann et al., 2003; Schubert, 2005; Kanemura et al., 2013). Furthermore, SEA also had a positive predictive value of 14% for developing seizures in a group of 347 ADHD children (Richer et al., 2002). It should be noted that SEA was only seen in some of the patients after photic stimulation or hyperventilation in the study of Richer et al. (2002).

**TABLE 4:** Prevalence of subclinical epileptiform activity (SEA) in attention-deficit hyperactivity disorder (ADHD).

References	N	Study design	EEG type	ED (%)	Localization
Kanemura et al. (2013)	46	Prospective cohort	Routine + photic stim 20 min	34.8	100% focal
Lee et al. (2016)	180	Retrospective	Routine	16.1	8.3% general 7.7% focal–frontal, Rolandic
Hughes et al. (2000)	176	Prospective	Routine 1 h with stimulation	30	24% focal 13% bifrontal
Hemmer et al. (2001)	234	Retrospective	Routine awake	15.4	60% focal, (5,6% Rolandic overall)
Millichap et al. (2011)	612	Retrospective	Routine	26.1	42.9% focal 41.7% generalized
Richer et al. (2002)	347	Retrospective	Routine 20 min + photic stim	6.1	–
Zaimoglu et al. (2015)	148	Prospective	Routine 1 h wake–sleep	26.4	Frontal, centrotemporal
Silvestri et al. (2007)	42	Prospective cohort	Sleep EEG (polysomnograpy)	53.1	28.2% centrotemporal, 12.5% frontal
Matoth et al. (2002)	126	Prospective cohort	Routine with stimulation	5	–
Socanski et al. (2010)	517	Retrospective cohort	Routine	7.5	53.9% generalized, 41% focal, 1.7% Rolandic

*N, number of patients; ED, epileptiform discharge.*

In Table 4, we collected the most recent studies on prevalence of epileptiform events. The study of Silvestri et al. (2007) reported the highest prevalence of SEA (53.1%) in their prospective cohort of 42 patients. It is noteworthy that this was the only study that used polysomnography for the evaluation. SEAs are known to be more frequent during sleep, however, the capture of these abnormalities is extremely problematic on a routine 20–30 min long EEG. It is an open question whether or not the subgroup of ADHD patients with SEA would benefit from AEDs by preventing progression of disease and decline of cognitive function. To conclude, several studies have suggested that EEG can be used in specific populations to exclude more crude pathology, albeit others did not support this view (Hemmer et al., 2001; Matoth et al., 2002; Socanski et al., 2010; Millichap et al., 2011). Clearly, there is an important role of investigating SEAs in central nervous system (CNS) pathology, such as ADHD. Until a consensus emerges, there is much room to expand further research.

## Mechanism of Cognitive Impairment

### Excitotoxity-Mediated Neurodegeneration

Neurodegeneration is a progressive loss of function and structure of neural cells leading to the death of neurons and glial cells (Spillantini and Goedert, 2013). The progressive decline of cognitive functions in neurodegenerative disorders is in line with the spreading of the accumulated misfolded proteins that is the major neuropathological hallmark of these disorders. The toxic proteins are different in the various forms of dementia (taupathies, amyloidopathies, synucleinopathies, etc.), however, they all have harmful effect on cellular membranes, mitochondrial functions, axonal transport, synaptic strength, and on neural survival in oxidative stress (Taylor et al., 2002). Misfolded proteins also change the physiological neuroinflammatory processes activating proinflammatory and neurotoxic mediators (Giovannini et al., 2002). As a summary of induced changes, protein misfolding associates with rapid neuronal death. Spatial distribution of pathological proteins varies among neurodegenerative disorders leading to different clinical presentations (e.g., entorhinal cortex is first to degenerate in AD, and substantia nigra is first in DLB and Parkinson’s disease). In MS, neurodegeneration also occurs in an interaction with autoimmune inflammatory responses targeting myelin and oligodendrocytes (Ellwardt and Zipp, 2014). Neurodevelopmental factors ending in decreased neural survival are crucial in the pathogenesis of ASD because of genetic mutations of synaptogenic, inflammatory moderator and axon mobility factors (Kalkan et al., 2016; Rani, 2019). Some studies demonstrated that neurodegeneration occurs in epilepsy, too (Frantseva et al., 2000; Rao et al., 2006). While the typical histopathological hallmark of temporal lobe epilepsy is the neural loss and gliosis detected in the hippocampus, amygdala, and entorhinal cortex, novel examinations report the presence of misfolded tau and amyloid proteins as well (Tai et al., 2016). Furthermore, neuroimaging and physiology data show progressive gray matter atrophy in the structures of epileptic network (Bernhardt et al., 2010).

A common feature among epilepsy and all neurodegenerative disorders is the increased cortical excitability (Di Lazzaro et al., 2004; Gilbert et al., 2004; Takarae and Sweeney, 2017). Growing body of evidence supports that increased excitability precedes neurodegeneration in various diseases. Vucic and Kiernan (2006) and Vucic et al. (2008) reported reduced short-interval intracortical inhibition prior to the symptom onset in patients with amyotrophic lateral sclerosis and with other motoneuron disorders using transcranial magnetic stimulation. According to the studies of Vossel et al. (2013), the occurrence of seizures is increased years before the initial symptoms of AD.

Elevated cortical excitability might contribute to neurodegeneration through excitotoxicity (Mehta et al., 2013). It refers to a toxic effect, resulting from prominent and prolonged activation of excitatory neural receptors causing cell death (Bano et al., 2005). Under normal conditions, glutamate acting on its postsynaptic receptors [*N*-methyl-D-aspartate (NMDA), α-amino-3-hydroxy-5-methyl-4-isoxazolepropionic acid (AMPA)] causes depolarization and permits the increase in intracellular calcium. If depolarization is prolonged or glutamate reaches an excessive concentration in the synaptic cleft, it turns into a neuron-killing toxin causing the disruption of cellular osmotic equilibrium (Ong et al., 2013).

**FIGURE 1:**
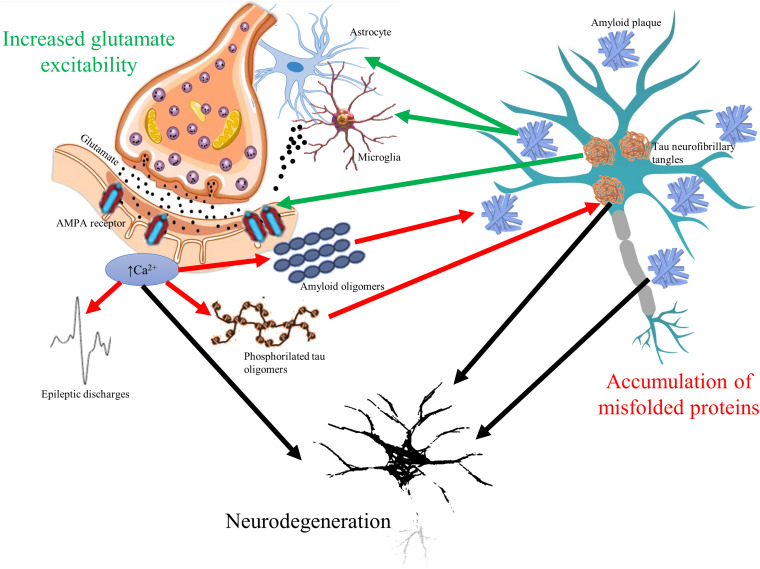
The vicious circle of glutamate mediated hyperexcitability and accumulation of misfolded toxic proteins in cognitive disorders. Glutamate neurotransmitter is altered in all cognitive disorders resulting in overexpression of α-amino-3-hydroxy-5-methyl-4-isoxazolepropionic acid (AMPA) receptors and elevated intracellular calcium signaling. Elevated calcium signal associates to higher release of amyloid oligomers to extracellular space and to increased phosphorylation of tau oligomers (red arrows). Increased firing of neurons represented by epileptic discharges is a consequence of glutamate-related hyperexcitability as well. On the other hand, accumulation of amyloid plaques and tau neurofibrils change glutamate receptor expression and induce excessive release of glutamate from microglial cells and astrocytes (green arrows). The bidirectional pathologic relationship could result in progressive neurodegeneration (black arrows), which is common hallmark of cognitive disorders.

The glutamate neurotransmitter system is affected in many diseases with cognitive symptoms (Figure 1). High level of calcium permeable AMPA receptors was identified in amyotrophic lateral sclerosis (Leal and Gomes, 2015). Inflammation induced, microglia driven excitotoxicity is a central event in MS (Gonsette, 2008). Elevated cortical glutamate concentration is a common finding in ASD (Brown et al., 2013). In AD, amyloid induces excessive glutamate release from astrocytes (Esposito et al., 2013), blocks the glutamate transporters of astrocytes responsible for reuptake (Zott et al., 2019), elevates calcium influx with the increase in depolarization (Fu et al., 2012), and activates NMDA receptors (Ferreira et al., 2010). Furthermore, tau might enhance the presynaptic glutamate release (Decker et al., 2016). Normal apoE function is essential in the attenuation of glutamate effect; however, its genetic mutation is the most known risk factor of AD (Aono et al., 2002). On the other hand, prolonged activation of NMDA receptors results in elevated production and secretion of amyloid-beta (Lesné et al., 2005) and in hyperphosphorylation of tau (Liang et al., 2009). It might explain the elevated phospho-tau level in surgical samples of temporal lobe epilepsy patients (Tai et al., 2016).

Since epileptic activity associates to excessive stimulation of NMDA receptors, it is intriguing to speculate that epileptic seizures might contribute to the neural loss presented in various forms of cognitive disorders. Indeed, the presence of epileptic seizures associates with faster progression of symptoms in AD (Vossel et al., 2013). However, short-lasting excitations (IEA and SEA) also associate with increased glutamate release (Kang et al., 2005), so harmful effect of epileptic discharges is predictable. It is reinforced by a study of Dolev et al. (2013), showing that even a 20 Hz burst activity could increase amyloid burden; by the study of Bero et al. (2011), showing that neuronal hyperactivity associates to increased amyloid burden; and by a report of Vossel et al. (2016), demonstrating the role of SEA in the accelerated progression of AD.

### Remodeling of Neural Circuitry

**FIGURE 2:**
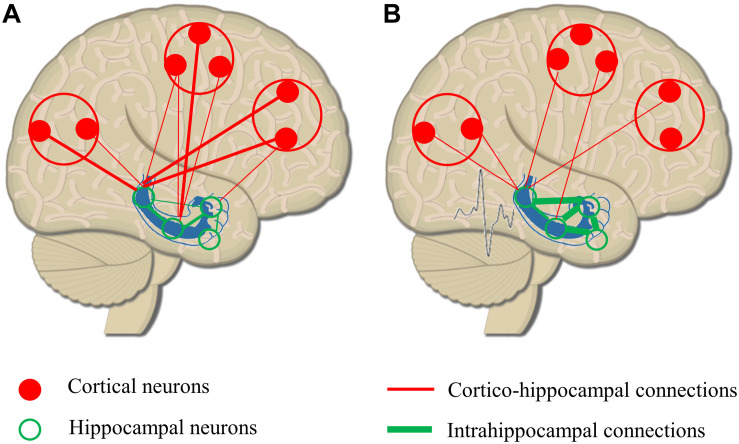
Remodeling or hippocampo-cortical circuitry as a result of epileptic discharges. **(A)** Physiological organization of hippocampo-cortical connections with numerous, strong local connections and less and weaker distant associations. **(B)** As a result of epileptic discharges, intrahippocampal connectivity is increased, and the strength and number of long distant connections are decreased. The remodeling of network circuitry leads to a relative isolation of hippocampus from cortical areas reducing the efficacy of hippocampo-cortical coupling.

Balance between excitatory glutamatergic and inhibitory GABAergic activity in the large functional networks of the brain is crucial in all cognitive functions (Sengupta et al., 2013). Reduction in inhibition or increase in excitation has a key role in ictogenesis (Bonansco and Fuenzalida, 2016). Local GABAergic sprouting limits the spreading of epileptic activity to distant areas (Sutula, 2002), relatively disconnecting the epileptogenic zone from connected brain structures. Connectivity studies support the pathological findings describing increased intrahippocampal and decreased hippocampo-cortical connectivity in patients with mesio-temporal lobe epilepsy (Warren et al., 2010; Engel et al., 2013). As seizures propagate and the epileptic network extends, altered hippocampo-cortical structural connectivity could lead to less synchronized global networks, to impaired organization of rhythmic brain activities, and finally to random organization of physiological networks (Luo et al., 2012; Figure 2).

Growing body of evidence suggests that IEA spreads in the same pathological network as epileptic seizures, however, the underlying aberrant activity does not reach the seizure threshold (Dzhala and Staley, 2003). This hypothesis is supported by clinical observations of transient cognitive impairment (TCI) observed after IEA. TCI is characterized by a brief temporary deficit in memory encoding, attention, communication, or visuospatial abilities (Holmes and Lenck-Santini, 2006). If IEA is frequent, epileptic activity could induce long-lasting and distant changes in brain functioning (Caciagli et al., 2014). It is supported by the findings of Gelinas et al. (2016) showing that IEA shows a coupling with spindles via cortical downstates. Studies using functional MRI reinforced these suggestions, demonstrating spike-related changes in blood–oxygen-level-dependent imaging (BOLD) signal even at distant cortical sites (Federico et al., 2005). Reports applying EEG connectivity analysis revealed similar findings demonstrating that functional connectivity is increased in the epileptic network during IEA similarly to seizure-related alterations, while it is reduced between epileptic and functional networks such as default mode network (DMN) (Bettus et al., 2008; Fahoum et al., 2013). Noticeably, these changes become permanent in a long-lasting disease and remain independent from ongoing IEA (Luo et al., 2011).

Substantial alterations of large neural networks have been shown in all conditions. Elevated intrahippocampal activity is depicted in the prodromal stages of AD (in amnestic mild cognitive impairment) (Bakker et al., 2012) correlating with cortical thinning (Putcha et al., 2011) and with disconnection to other neural networks including DMN (Pasquini et al., 2015). Similar findings showing local increase in connectivity and reduction in global connectivity have been described in various forms of dementia including DLB and FTD (Agosta et al., 2013; Dauwan et al., 2016). Hyperactivity within large-scale brain networks and decreased between-network connectivity is a core finding in pediatric ASD studies (Cerliani et al., 2015; Nomi and Uddin, 2015). Increased within-network hyperconnectivity has been described in the frontal areas of ADHD patients (Wang et al., 2009) with a loss of long distant connections. Interestingly, AD-like changes in hippocampo-cortical connectivity (increase in intrahippocampal connectivity and decrease in global connectivity) have been demonstrated in MS patients with memory impairment; however, they parallelly identified reduction in hippocampal activation (Hulst et al., 2015).

### Disruption of Sleep-Related Memory Consolidation Process

Sleep occurs in all vertebrates in regular intervals, and it is homeostatically regulated. It is well known that sleep deprivation has a harmful effect on the physical and mental health including severe changes in hormonal, homeostatic, and temperature regulation, higher occurrence of infections, and dysfunction of cardiovascular control (Shahar et al., 2001). Human sleep is distinguished into non-REM and REM sleep. The dual process hypothesis postulates that REM sleep is crucial in implicit memory formation, while non-REM sleep, especially episodes characterized by slow-wave electric activity (slow-wave sleep or SWS) is mandatory in the establishment of episodic memory (Diekelmann and Born, 2010). The widely accepted two-stage memory model differentiates brain structures into areas with short-term memory capacity having an encoding function and into regions serving as long-term storages (Walker, 2005). The memory consolidation process involves the repeated reactivation of short-term stored memory items (freshly developed synaptic connections) during offline periods (e.g., SWS) and the strengthening and adaptation of memory fragments into long-term storages (Stickgold, 2005).

The anatomical structure for the interplay is the network between hippocampus and cortical areas. In human SWS, EEG shows 0.5–4 Hz slow oscillations with dynamic alterations of neuronal membrane depolarization (upstates) and hyperpolarization (downstates) (Csercsa et al., 2010). Dynamic changes reveal an opportunity for the reduction in weaker synaptic connections parallel with the reinforcement of stronger ones, known as synaptic downscaling (Tononi and Cirelli, 2006). Neurons during SWS show widespread synchronization in cortico-cortical, thalamo-cortical, and hippocampo-cortical networks (Dang-Vu et al., 2008). High synchrony is reinforced by animal and human neurophysiology studies showing that the top–down controlled phase-locked co-occurrence of hippocampus generated sharp-wave ripples, thalamic sleep spindles, and cortically induced slow waves (Maingret et al., 2016).

**FIGURE 3:**
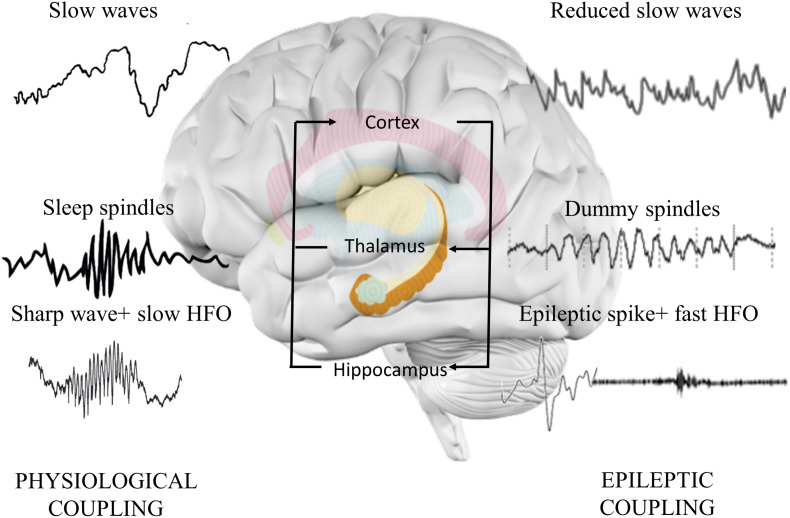
Hippocampo-thalamo-cortical coupling in memory consolidation. In physiological memory consolidation process, synchronization of the hippocampus, thalamus, and neocortex is essential. Hippocampal sharp-wave ripples correspond to the replay of recently stored memory items in the synaptic connections of hippocampal neurons. Thalamic sleep spindles with a frequency of 12–16 Hz are essential elements of memory formation, synchronizing hippocampal activity with cortical neurons. Cortical sleep-related slow waves provide the highest synchronization state to facilitate the activation of hippocampal sharp-wave ripples and thalamic sleep spindles. Epileptic discharges correspond to the pathological transformation of sharp waves coupling with faster high frequency oscillations. The altered activity disorganizes the architecture of spindles, decreases the normal spindle activity, and induces the formation of dummy spindles with longer duration and spiky appearance. Cortical slow waves are also reduced, probably due to the spike-inducted cortical hyperpolarization (downstates). Alterations might reduce the efficacy of memory consolidation process.

An epileptic spike is shorter but similar to sharp wave, and it associates to faster ripple oscillations than sharp wave (Bragin et al., 2002). Numerous studies hypothesized that epileptic discharges linked to fast ripples could interfere with normal memory process (Halász et al., 2019; Figure 3). Furthermore, they can also act as dysfunctional (“dummy”) variants of sharp-wave deteriorating memory consolidation (Gelinas et al., 2016). The crucial role of sleep-associated IEA in memory formation is suggested by the following findings: IEA predominantly occurs in SWS (Bazil, 2000); it associates with longer REM latency (first occurrence of REM during the night), with reduced duration of SWS (Miller et al., 2016) and with lower number of physiological ripples (Jefferys et al., 2012) and negatively affects thalamic spindle formation (Frauscher et al., 2015).

While sleep changes might have a crucial role in the memory impairment of epileptic patients, sleep disorders are also highly prevalent in cognitive disorders. Around 40% of AD patients suffer from sleep disturbances (Tractenberg et al., 2003), namely, from nocturnal sleep disruption, increased daytime sleepiness, insomnia (Rao et al., 2008; Osorio et al., 2011), and sundowning (agitation and confusion late afternoon) (Volicer et al., 2001). About 50–83% of DLB patients suffer from REM sleep behavior disorders (Ferman et al., 2010). In MS, obstructive sleep apnea (Braley et al., 2014), restless leg syndrome (Manconi et al., 2007), and moderate or severe insomnia are frequently observed (Brass et al., 2014). Insomnia is reported in 44–83% of children with ASD (Miano and Ferri, 2010). Prominent elevation in the occurrence of restless leg syndrome, periodic limb movement in sleep, sleep-onset insomnia, nocturnal motor activity, and obstructive sleep apnea has been highlighted in numerous studies on ADHD patients (Konofal et al., 2010). Sleep microstructure seems to be highly impaired as well in cognitive disorders. Reduced REM sleep, decreased number of sleep spindles, reduction in SWS, and increase in superficial stages have been reported in ASD (Richdale and Schreck, 2009). Excessive loss of SWS is a characteristic hallmark of AD with a reduction in sleep spindles and K complexes (Petit et al., 2004). Children with ADHD show lower rate of cyclic alternating pattern and sleep spindles (Miano et al., 2006; Kirov and Brand, 2014). Thus, the role of poor sleep in cognitive impairment is not questionable in cognitive disorders.

While SWS is reduced in all cognitive disorders, SEA seemingly still accumulates in deep sleep similarly to epileptic patients. Approximately 90–100% of epileptic discharges are detected in SWS in studies examining patients with AD (Vossel et al., 2016) or with ASD (Chez et al., 2006). Furthermore, the occurrence of epileptiform discharges on nocturnal EEG is positively related to higher attention deficit and higher impulsivity in ADHD patients (Danhofer et al., 2018). Since epileptic activity compromises the organization of sleep structure and disturbs the sleep-related memory consolidation processes, it is intriguing to state that SEA might accelerate the disorganization of sleep structure and contribute to the decline of memory functions.

## Therapeutic Aspects

### Current Findings and Recommendations

The primary application of AEDs is to effectively reduce or eradicate epileptic seizures with an optimal side effect profile. Approximately 30 types of AEDs are available on the market with first, second, and third lines of indications regarding the type of seizures, the age, physical condition, and the current drug use of the patient. While we do not understand completely the mechanism of all AEDs, their efficacy is measured as the extent of decrease in the number of seizures. While we have tremendous experience and recently updated guidelines for controlling seizures in epilepsy patients, we have relatively limited data on the AED selection in cognitive disorders. In AD, studies are available on levetiracetam (LEV), lamotrigine (LTG), gabapentin, carbamazepine, valproic acid, phenytoin, and phenobarbital (Horváth et al., 2016; Vossel et al., 2017). Only LEV and LTG reached excellent efficacy (60–70% reduction in the number of seizures in a 1-year follow-up) and tolerability without cognitive side effects (Belcastro et al., 2007; Cumbo and Ligori, 2010; Lippa et al., 2010). Notably, treatment with LEV resulted in marginally increased cognitive scores (MMSE and ADAS-Cog), and application of LTG was associated with significantly improved mood (Cumbo and Ligori, 2010), but the study was not placebo controlled. Studies on AED application in other NCDs for controlling seizures are absent (Horváth et al., 2018a). According to the current guidelines, the management of seizures does not differ in other cognitive disorders compared to epilepsy patients (Myers and Johnson, 2007; Kelley and Rodriguez, 2009; Felt et al., 2014).

While growing body of evidence supports the central role of epileptic discharges in cognitive deterioration, studies on affecting SEA are limited in cognitive disorders. In NCDs, LEV treatment for 2 weeks significantly improved performance in pattern separation, but no other cognitive scores of non-epileptic MCI patients in line with the normalization of hippocampal and entorhinal cortical activity measured with functional MRI (Bakker et al., 2015). In the study of Musaeus et al. (2017) using single-dose LEV, while antiepileptic therapy marginally increased the power of beta band in AD patients, positive cognitive effect was not detected. Unfortunately, studies on other forms of NCDs have not been conducted. Furthermore, most MCI and AD studies are not double-blind observations and did not use SEA as a selection criterion or a marker of therapeutic response. Ongoing clinical trials (e.g., ILiAd, NCT03489044; LAPSE, NCT04004702; LEV-AD, NCT02002819 studies) on LEV already assess SEA for the identification of target groups, but results have not been published yet. In ASD patients without epileptic seizures, seven placebo-controlled, randomized studies on the use of AED are available. These studies analyze the utility of valproic acid, topiramate, LTG, and LEV. Based on the findings of a meta-analysis, AED did not have a significant effect on behavioral symptoms, however, studies have not differentiated subgroups of patients with SEA and were not EEG controlled (Hirota et al., 2014). In ADHD, only independent, single reports are available on the use of AED in non-epileptic patients. A study using valproic acid reported reduction in frontal SEA in 62% of ADHD patients, and the decrease was correlated with improvements in ADHD rating scale (Kanemura et al., 2013). In the study of Öncü et al. (2014), LTG improved mood scores in 78% of ADHD patients with comorbid bipolar disorder or depression, however, EEG was not applied. While AED are frequently prescribed in MS for neuropathic pain, studies on SEA or on cognitive impact were not conducted (Solaro et al., 2005).

Age-related changes in pharmacodynamics and pharmacokinetics make AED studies by the elderly complicated. In the selection of AEDs, safety issues and contraindications have to be carefully considered in these patients. In previous studies, the use of AEDs has been associated with elevated risk for fall (Seppala et al., 2018), stroke (Sarycheva et al., 2018), fractures (Shen et al., 2014), pneumonia (Taipale et al., 2019), and adverse drug–drug interactions (Anderson, 2004). Application of traditional AEDs (e.g., phenytoin, valproic acid) was associated with unplanned hospital admissions and impaired motor functions (Lin et al., 2017). Use of carbamazepine and oxcarbamazepine was attached to adverse cardiac events, hyponatremia, and sedation (Spina and Perucca, 2002). Thus, contraindications have to be considered individually.

For the understanding of the potential role of AED in the therapy of cognitive impairment, double-blind, placebo-controlled studies are needed in various cognitive disorders. The detection of SEA with EEG might have a crucial role in the accurate identification of target groups of patients, and it might serve as a fundamental outcome and therapeutic response measure. However, it should be noted that AEDs are primarily applied for seizure control. Thus, their effect on inhibiting epileptiform discharges (including IEA or SEA) is limited or unknown. Furthermore, the identification of novel targets and development of new drugs are crucial for the proper therapy of hyperexcitability in cognitive disorders.

### Potential Novel Directions

As we have described, many AEDs have been introduced to the market over the recent decades and even more are in development. Fundamentally, all AEDs have been designed or optimized to restore an abnormal balance between excitatory and inhibitory neurotransmission, which is a hallmark of epilepsy. Most AEDs, especially those from the first generation, lack selectivity and act on essential mediators of neuronal excitability such as ion channels, glutamate, or GABA_A_ receptors. These drugs exert widespread effects on neuronal networks and cause a range of undesired side effects such as sedation and cognitive deficits (Ortinski and Meador, 2004). Therefore, newer antiepileptics that have more selective targets modulating excitability in discrete neuronal circuits are better positioned for potential treatment of SEA or IEA associated with various neuropsychiatric disorders.

One such drug with a unique modulatory effect on neuronal excitability exerted by binding to the synaptic vesicle protein 2A (SV2A) is LEV (Löscher et al., 2016). Compared to more conventional AEDs, which typically act on postsynaptic receptors or ion channels, levetiracetam tends to be better tolerated by patients and does not induce strong sedative effect (Cramer et al., 2003; Abou-Khalil, 2008). Interestingly, levetiracetam has been initially developed as a cognitive enhancer after chemical modification of its predecessor, piracetam. Several lines of evidence indicate that low doses of levetiracetam improve cognitive performance in both animal models and clinical setting. These therapeutic activities of the drug are attributed to modulation of hippocampal hyperactivity (Haberman et al., 2017). Interestingly, levetiracetam is one of the few AEDs that display clear-cut effect on IEA not only in patients with adult (Stodieck et al., 2001) and childhood epilepsies (Larsson et al., 2010), but also in children with ADHD (Bakke et al., 2011) resulting in improvement in clinical symptoms (e.g., restless leg) (Gagliano et al., 2011). Further, preclinical evidence indicates that levetiracetam improves cognitive performance in models of AD and schizophrenia (Sanchez et al., 2012; Koh et al., 2018). There may be a connection with the mechanisms of action of levetiracetam since several studies using SV2A PET tracers show reduction in SV2A expression associated with several neuropsychiatric and neurodegenerative diseases that are associated with cognitive deficits (Heurling et al., 2019). This was most clearly demonstrated in patients with AD (Chen et al., 2018). It is believed that SV2A is a marker of synaptopathy reflecting pathological changes in synaptic circuits (e.g., hippocampus) associated with cognitive performance. These observations have led to a number of clinical trials exploring the potential of levetiracetam as treatment for cognitive deficits associated with increased cortical activity or with SEA in AD (Bakker et al., 2015; Vossel et al., 2016), and numerous ongoing double-blind trials are going to conclude soon as well.

A promising potential therapeutic approach can be attributed to subunit selective modulators of GABA_A_ receptors, which have discrete localization in the brain areas associated with SEA or IEA. The key advantage of such compounds is their improved safety and tolerability versus conventional, non-selective drugs such as benzodiazepines. In this context, selective positive allosteric modulators of alpha-5 subunit containing GABA_A_ receptors might have a potential to reduce the occurrence of epileptiform discharges (Biagini et al., 2010), and studies have shown promising effects on cognitive and memory performance in animal models (Koh et al., 2013).

Abnormality in glutamate uptake is another important mechanism that is shared by several neuropsychiatric diseases that are associated with SEA and deficits in cognition (O’Donovan et al., 2017). Recent work indicates that neuronal hyperexcitability observed in the limbic regions in patients with Alzheimer’s disease may be initiated by suppression of glutamate reuptake and can trigger a vicious cycle of neurodegeneration driven by β-amyloid (Zott et al., 2019). Therefore, restoration of glutamate uptake by drugs increasing the expression or function of excitatory amino acid transporter 2 (EAAT2) could find a novel therapeutic indication for treatment of SEA or IEA associated with various cognitive disorders (Fontana, 2015).

## Discussion

Cognitive disorders including NCDs, ASD, ADHD, and MS have a high overall prevalence affecting approximately 40–50% of the population. A common hallmark of these variable conditions is the higher occurrence of epileptic seizures during the course of the disease suggesting that hyperexcitation might play a role in the pathomechanism of cognitive impairment (Tuchman and Rapin, 2002; Austin and Caplan, 2007; Horváth et al., 2016). Prevalence and impact of IEA and SEA are less investigated in cognitive disorders in comparison to epileptic seizures. However, the study of these phenomena might represent an important future direction, since modern epileptology recognized that isolated but frequent epileptic activity could compromise the cognitive function of epilepsy patients more than epileptic seizures (Berg, 2011).

Proper definition and/or distinction of IEA and SEA are also missing, making it difficult to compare the results of various prevalence studies. While the interictal terminology postulates the presence of ictus (seizure), IEA is frequently used to describe epileptiform activity without overt clinical seizures. However, traditional epileptology recognizes epileptiform discharges without detectable clinical or electrographic seizures as benign EEG variants (Santoshkumar et al., 2009). From the epileptological viewpoint, benign means that the detected activity does not associate to clinically diagnosed epilepsy or any other neurological or psychiatric disorder. However, in our opinion, independency from seizures does not necessarily equal to clinically benign behavior. A possible explanation is that epileptiform discharges and epileptic seizures are consequences and markers of increased cortical excitability, however, they represent the different ends of the spectrum (Badawy et al., 2009a). If network excitability exceeds a certain threshold, the affected patient develops epileptic seizures and frequent interictal epileptic discharges, leading to the diagnosis of epilepsy (Dzhala and Staley, 2003). If it does not reach the threshold, SEA is detectable and indicates increased excitability as a general marker (Badawy et al., 2009b). Since more and more neuropsychological and neuroimaging studies suggest that SEA correlates with cognitive deterioration (Vossel et al., 2016), we propose to reconsider the use of “benign” term for epileptiform EEG graphoelements without detailed neuropsychological investigation. In our review, we systematically separated the two terms, IEA and SEA, and propose the exclusive use of SEA for epileptiform events in the absence of proved epileptic seizure. However, the distinction resulted in an important conclusion: SEA shows the similar characteristic as IEA regarding the temporal, spatial characteristic, and the impact on cognitive functions.

IEA and SEA both accumulate in sleep in AD patients (Vossel et al., 2016; Horváth et al., 2017a), in ASD patients (Chez et al., 2006), and in ADHD (Silvestri et al., 2007). IEA and SEA both recorded mainly over the frontotemporal areas in AD (Rao et al., 2009; Vossel et al., 2013, 2016; Cretin et al., 2016; Sarkis et al., 2016; Horváth et al., 2018b), in ADHD (Lee et al., 2016), and in ASD (Chez et al., 2006). Frequent occurrence of interictal discharges associate to decreased therapeutic response, poorer postsurgical outcome, and augmented cognitive decline in epilepsy patients (Drane et al., 2016). Higher frequency of IEA in SWS defines more prominent impairment in language function of epileptic patients with ESES (Scheltens-de Boer, 2009). Therapeutic reduction in IEA improved the behavioral problems of children with focal epilepsy (Pressler et al., 2005). SEA is attached to two times faster progression of AD (Vossel et al., 2016), higher prevalence of regression in ASD (Giannotti et al., 2008; Stefanatos, 2008), elevated number of active lesions in MS (Lebrun, 2006), and higher incidence of future seizures in ADHD (Lee et al., 2016). Reduction in SEA by AED led to 60% improvement in behavior scores in ADHD patients (Bakke et al., 2011), improved cognitive scores in patients with mild cognitive impairment (Bakker et al., 2015), and significant positive changes in cognitive scores of ASD patients (Hollander et al., 2001).

Based on literature overview, there are various ways how SEA could have a detrimental effect on cognition. The common link is the glutamatergic system that is compromised in all cognitive disorders (Gonsette, 2008; Esposito et al., 2013). Increased excitatory activity results in related excitotoxicity leading to neurodegeneration of various neural structures in different cognitive disorders (Mehta et al., 2013). In the neurodegenerative process, epileptic discharges accelerate the accumulation of toxic proteins (e.g., tau, amyloid) facilitating neural loss (Liang et al., 2009; Dolev et al., 2013). These processes transform into a vicious circle since misfolded proteins also induce excessive release of glutamate (Ittner and Götz, 2011). The described changes lead to a local hyperexcited neural network and activation of compensatory remodeling mechanisms (Sutula, 2002). Remodeling can lead to the disconnection of the affected structures to other functional networks (Engel et al., 2013). It is supported by numerous studies demonstrating the loss of distant connections in cognitive disorders (Liu et al., 2014). Extending general excitability might have a crucial role in the spreading of pathological proteins in the functional neural networks of patients with NCDs (Pievani et al., 2011; Hoenig et al., 2018). When large functional circuits are involved in the pathological process, their function shows abnormalities, and it is demonstrated by the alteration of sleep structure (Palop and Mucke, 2010). Increasing number of epileptic discharges leads to reduction in slow-wave sleep, overproduction of dysfunctional, dummy sleep spindles, and finally loss of sleep function in the memory encoding and consolidation process (Malow, 2007; Halász et al., 2019). Since epileptic discharges are crucial in the pathological process, modification might have a novel therapeutic potential in cognitive disorders (Bakker et al., 2015).

Rapidly emerging observations and data linking SEA or IEA with a wide range of neuropsychiatric and cognitive disorders open several previously unexplored therapeutic opportunities that could be focused on targeting neuronal hyperexcitability. In this context, an obvious solution would be application of existing AEDs, which should be able to normalize such abnormal neuronal activity. However, despite some promising results with selected AEDs, this class of drugs is generally associated with poor tolerability, narrow therapeutic window, and worsened cognitive abilities. Therefore, a more selective and perhaps milder modulation of neuronal excitability in discrete brain regions in stratified subpopulation of patients with documented SEA or IEA could lead to significant therapeutic benefits and become a novel class of therapy for cognitive disorders.

## Author Contributions

AH and EC conceived the topic and wrote the manuscript. SL, RK, and AK contributed to writing and reviewing and editing the manuscript. All authors contributed to the article and approved the submitted version.

## Conflict of Interest

The authors declare that the research was conducted in the absence of any commercial or financial relationships that could be construed as a potential conflict of interest.
